# Get the message? A scoping review of physical activity messaging

**DOI:** 10.1186/s12966-020-00954-3

**Published:** 2020-04-15

**Authors:** Chloë Williamson, Graham Baker, Nanette Mutrie, Ailsa Niven, Paul Kelly

**Affiliations:** Physical Activity for Health Research Centre (PAHRC), Institute for Sport, Physical Education and Health Sciences, University of Edinburgh, Edinburgh, UK

**Keywords:** Exercise, Public health, Guidelines, Communication, Dissemination

## Abstract

**Background:**

Understanding how to create and deliver effective physical activity (PA) messages for and to various population subgroups may play a role in increasing population PA levels. This scoping review aimed to provide an overview of what is known about PA messaging and highlight key research gaps.

**Methods:**

We followed a 5-stage protocol proposed by Arksey & O’Malley and the Preferred Reporting Items For Systematic Reviews and Meta-Analyses (PRISMA) extension for scoping reviews checklist. *Stage 1*: research questions were identified. *Stage 2:* we identified relevant studies by searching electronic databases, contacting existing networks and hand searching reference lists. *Stage 3*: studies were screened in Covidence™ software. *Stage 4*: study data were extracted and charted. *Stage 5*: findings from included studies were collated, summarised and reported in two ways: (1) a descriptive numerical analysis providing insight into extent, nature and distribution of the included studies, and (2) a narrative summary summarizing the evidence reviewed organised by messaging concepts and by population subgroup.

**Results:**

A total of 9525 references were imported into Covidence™ for screening. Of these, 123 studies were included in final analysis. We found that PA messaging evidence is complex and multidimensional in nature, with numerous concepts to consider when creating or evaluating messages. The extent to which these different PA messaging concepts have been researched is variable. Where research has accumulated and evidence is consistent, it supports the following: (1) PA messages should be framed positively and highlight short-term outcomes specifically relating to social and mental health, (2) message content should be tailored or targeted to intended recipient(s), and (3) when developing messages, formative research, psychological theory and/or social marketing principles should be used.

**Conclusion:**

While it is unlikely to address global inactivity on its own, PA messaging may play a valuable role improving population PA levels. However, it is a complex and multidimensional concept and greater understanding is still needed. We present a synthesis of the existing evidence, highlighting key areas where evidence has accumulated and where gaps lie, as well as recommendations for PA messaging to different population subgroups.

## Introduction

Physical inactivity is a major contributor to the growing global burden of non-communicable diseases including cancer, cardiovascular disease, depression and diabetes [1]. Recent research shows that overall, global trends are worsening with physical inactivity levels rising in many high-income Western countries and with the steady prevalence of inactivity in low income countries making a substantial contribution to the burden of disease [2]. It has been suggested that a systems approach may play an important role in responding to complex public health challenges, such as efforts to increase PA at population level [3–7]. A systems approach acknowledges that alongside efforts to modify policy and the physical environment to promote PA [8] there is an important role for interventions which aim to address individual factors. One such approach is PA messaging.

Interventions utilising a PA messaging approach tend to feature the delivery of information to members of a target group within the public with the aim of either directly or indirectly improving PA levels. Understanding how to utilise PA messaging effectively is important for three reasons. Firstly, messaging is a scalable approach that can be used to reach large numbers of people at relatively low cost [9]. Secondly, effective messaging can augment the dissemination of PA guidelines and related information such as benefits of PA to various population subgroups, as this information is generally not created to be public facing or to motivate people to become or remain physically active [10]. Indeed, evidence suggests that the general public have limited knowledge of the current PA recommendations for health [11]. Thirdly, existing evidence shows PA messaging interventions to date have had limited effects on PA behaviour itself and mixed findings on outcomes such as awareness and motivation [9, 10, 12–18]. Further research to understand how to effectively develop and deliver PA messages for and to different population subgroups is therefore warranted.

In terms of previous research into PA messaging, a number of reviews exist. While these reviews have focused on specific aspects of messaging such as guideline dissemination [16, 17], solely on message content [10], specifically on mass media campaigns [9, 12, 13, 19], or only included research from the USA [18], no review that provides a broad overview of the evidence on PA message content and delivery from multiple countries across the globe has been conducted. A summary paper which maps and synthesises what is currently known across the various PA messaging concepts may be an important step in understanding how to optimally create and deliver effective PA messages for various population subgroups. Therefore, the aim of this study was to provide the first such broad scoping of the evidence on PA messaging. Specifically, we sought to answer the following research questions:

1. What is known about (a) PA message content and (b) PA message delivery?
2. What is known about PA message content and delivery for specific population groups?
3. What are the research gaps?

## Methods

### Study design and protocol

Based on the study aim, a scoping review was determined to be the most appropriate method. The aim was too broad to address via a traditional systematic review (and meta-analysis), and could be more appropriately answered through examining the extent, range and nature of research in this area, summarising and disseminating research findings to date, and identifying research gaps in this area; all of which are common scoping review purposes [20]. The process of conducting a scoping review is often iterative, allowing for changes to inclusion and exclusion critera, research questions and analytical approaches as more is learned about the evidence base. Furthermore, a scoping review allows for inclusion of a broad range of study designs, providing a more comprehensive picture of the research area. To ensure robustness, this study adopted an established five-stage scoping review protocol proposed by Arksey & O’Malley [20] and built upon by Levac, Colquhoun & O’Brien [21], and followed the Preferred Reporting Items For Systematic Reviews and Meta-Analyses (PRISMA) extension for scoping reviews checklist (Additional File 1) [22].

### Key definitions and position of messaging

Health communication encompasses the study and use of communication strategies to inform, influence and motivate individual, institutional and public audiences about important health issues [23]. At the outset of this review, we sought to define and position PA messaging within the wider context of health communication to inform searches and inclusion criteria. We present PA messaging as a subtype of health communication and as an overall concept that encompasses both content and delivery aspects of a PA message. As no universally used definition of PA messaging exists, working definitions were developed by the study authors for the purpose of this study (Table 1).

**Table 1:** Working definitions for the purpose of this research

Term	Working definition
Physical activity messaging	The overall process of designing, creating and delivering physical activity messages
Physical activity message	Educational or persuasive material to be relayed to a specific individual or group within the public with the aim of ultimately increasing physical activity levels
Physical activity message content	The specific aspects which comprise a PA message, such as the type, amount and presentation of information
Physical activity message delivery	The process by which a physical activity message is delivered to the target individual or group of the public

Definitions adapted from Latimer et al. [10] and drawing on Michie et al., intervention functions [24]

Initial literature searching to establish an understanding of key PA message and messaging terms revealed multiple sub-concepts and inconsistencies in the use of terminologies surrounding these. Indeed, these inconsistencies and the need to take caution when comparing studies have been previously noted [25]. One example of this are the terms *tailoring* and *targeting.* Although some authors clearly distinguish between tailoring as an exclusively individual level approach and targeting as an exclusively group level approach [16], the term tailoring has also been used to describe customisation of message content at an individual level [26], and at a group level [27]. Similarly, the term targeting has been used to describe a group-level approach [28] as well as to describe individually-customised messages [29]. Thus, a glossary of PA messaging sub-concepts and their working definitions for the purpose of this scoping review was created and is presented in Table 2. Establishing these working definitions was a fundamental step as it allowed us to standardise information from various studies despite inconsistencies in terminologies used and thus reliably extract data in Stage 4.

**Table 2:** Working definitions of key physical activity message content and delivery concepts

	Working definition
**Message Content**	
*Type of information*	The nature or purpose of information included in the message. Messages identified in the literature can generally be grouped into three broad categories: ‘how much and what type’ information (such as physical activity guidelines), ‘why’ information (such as benefits of physical activity), and ‘how to’ information (practical and supportive information).
*Use of gain- or loss- framing* [10]	The use of framing a message to highlight either the benefits of taking part in physical activity or the consequences of not taking part.
*Tailoring* [30]	Information based on individual user data (e.g. specific feedback on pre-established goals such as step counts)
*Targeting* [30]	Information designed to be relevant to a specific group (e.g. inactive individuals or diabetics)
*Personalisation* [30]	The use of static, user-specific information in a message (e.g. name or home address).
**Message Delivery**	
*Media or mode of delivery*	The type of media through which the message is being relayed, for example, emails, posters or radio adverts.
*Provider or source*	The provider or source of the message, for example, GP, the media, or friends and family.
*Frequency and dose*	How often the message is delivered and for how long, for example, emails sent 3 times a week for 4 weeks.

### Stage 1: identifying the research question

Our research aim was to provide a broad overview of what is known about PA messaging. To address this aim, three specific research questions were identified.

1. What is known about (a) PA message content and (b) PA message delivery?
2. What is known about PA message content and delivery for specific population groups?
3. What are the research gaps?

### Stage 2: identifying relevant studies

We identified relevant studies by:

1. Searching the following electronic databases: Ovid (MEDLINE), ProQuest, SPORTDiscus (Ebscohost), and Web of Science
2. Contacting existing academic, policy and practice networks requesting relevant studies
3. Hand searching reference lists of key studies and checking recent publications by key authors

The database search strategy was designed to be as comprehensive as possible with the available resources. Databases were searched for titles that contained at least one “PA” term as well as at least one “messaging” term (full list of search terms can be found in Supplementary Table 1, Additional File 2). Appropriate truncation symbols and wild cards were used to account for search term variations and maximise searches. No limits on journals searched were used. As an example, the full electronic search strategy for MEDLINE can be found in Supplementary Table 2, Additional File 2. Searches were conducted up to August 30th, 2019. Inclusion and exclusion criteria (see Table 3) were designed to be highly inclusive.

**Table 3:** Inclusion and exclusion criteria

Inclusion Criteria	Exclusion Criteria
• Research articles or reports in any geographical location or setting • Research conducted in healthy or clinical populations • Articles published in peer-reviewed journals and grey literature • Articles reporting on development of or effects of PA messages • Articles published in English • Research designs including: empirical research studies (qualitative, cross-sectional or longitudinal designs, interventions or natural experiments with pre-post measures or comparison) and non-empirical research (systematic and non-systematic reviews, and methods or theory papers)	• Articles focusing on wider PA communication not within the scope of this review, for example messages not directed to public or studies using other communication techniques such as one-to-one counselling • Abstracts without full text

### Stage 3: study selection

All identified studies were uploaded to Covidence™ software where duplicates were automatically removed at time of upload**.** Titles and abstracts were screened by CW with 15% double screened by either GB or PK. Full text level reviewing was carried out by two independent researchers (CW, PK, GB or AN) with conflicts resolved by a third researcher.

### Stage 4: charting the data

Data were extracted and entered into a data charting form using Excel. Where available, the data charted included all of the following:

- General study information including author, title, study location, study design and participant information
- Description of study and message used
- Primary focus of study (message content, delivery or both)
- The use or absence of psychological theory
- The use or absence of of social marketing principles

### Key findings

- Implications

### Stage 5: collating, summarising and reporting

In a scoping review, there are numerous ways in which data from identified studies can be organised, synthesised and reported. Findings of this scoping review were reported in two ways: (1) through a descriptive numerical analysis providing insight into extent, nature and distribution of the included studies, and (2) through a narrative summary of the evidence base. To address our research questions and maximise relevance for researchers, policymakers, practitioners and other relevant stakeholders, we made the decision to organise the narrative summary by the pre-identified messaging constructs of content and delivery (see Table 2) as well as by UK Chief Medical Officer’s guideline groups (Children and Young People, Adults, Older Adults, Pregnant Women and Disabled People) [31]. Finally, a matrix displaying where consistent research had accumulated and where gaps lay, organised by messaging concept and population group was created. This matrix displays research areas across 5 levels of evidence from strong (e.g. systematic review level evidence) to non-existent (i.e. no research identified in this review) based on the number, type and agreement of findings across studies relating to each research area.

## Results

### Descriptive numerical analysis

**Fig. 1:**
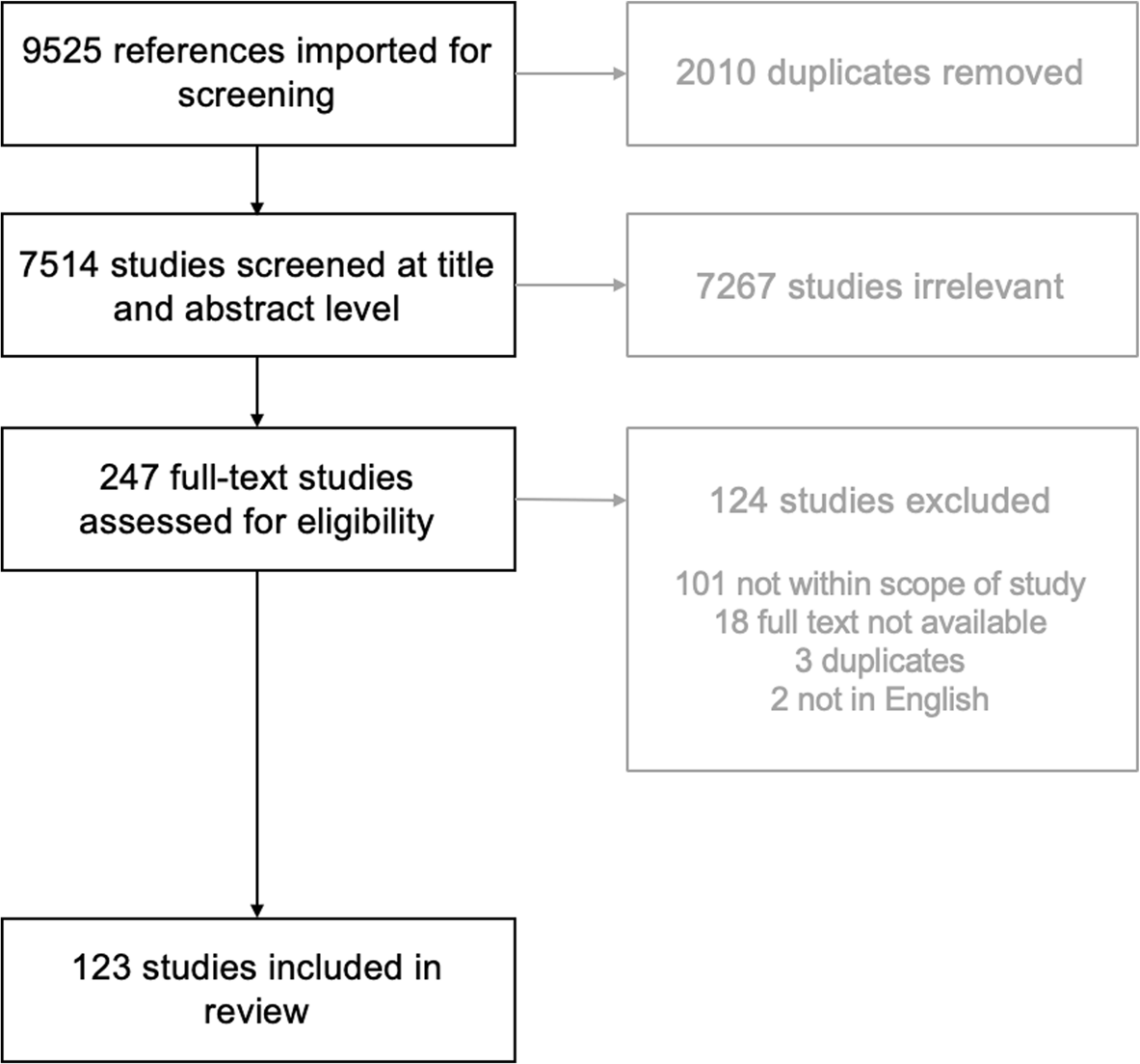
Study selection flowchart

A total of 9525 references were identified for screening (*n* = 9514 from database searches, *n* = 6 from existing networks, and *n* = 5 from hand searching). Following removal of duplicates and screening, 123 studies were included in final analysis. A study selection flowchart is presented in Fig. 1.

Of these 123 studies, 99 were empirical (original research) and 24 were non-empirical (reviews, commentary, methods, etc). Of the 99 empirical studies, there were 78 experimental studies (34 between-groups studies, 16 pre-post, 13 randomised controlled trials, 6 cross-sectional, 4 within-subjects, 3 quasi-experimental, 1 longitudinal, 1 post-test only, 1 single-group experiment, and 1 uncontrolled trial) and 21 non-experimental (18 qualitative and 3 cross-sectional) studies. Of the non-empirical studies, there were 13 non-systematic reviews, 6 systematic reviews, 3 commentaries, 1 conference foreword, and 1 methods paper.

The 99 empirical studies took place in the following locations: Canada (*n* = 34), USA (*n* = 32), Australia (*n* = 12), UK (*n* = 8), China (*n* = 2), France (n = 2), Germany (*n* = 2), Japan (*n* = 2), Belgium (*n* = 1), Brazil (*n* = 1), Ireland (*n* = 1), Netherlands (*n* = 1) and 1 took place in multiple countries (Bulgaria, Croatia and Romania). Almost half of the included studies (*n* = 58, 47.2%) were published in the past 5 years (2014–2019). The relevant guideline groups of the 123 studies were as follows: Adults (*n* = 61), Adults and Older Adults (*n* = 17), Children and Young People (*n* = 16), Children and Young People & Adults (*n* = 9), Pregnant Women (*n* = 4), Older Adults (*n* = 3), Disabled People (*n* = 3), and All (*n* = 10).

Of the 99 empirical studies, 62 (62.6%) stated use of psychological theory. The most commonly identified psychological theories were Theory of Planned Behaviour (*n* = 11), Social Cognitive Theory (*n* = 9), Self-Determination Theory (*n* = 5) and Elaboration Likelihood Model (*n* = 4). Authors in 8 of the 99 empirical studies (18.2%) reported use of social marketing principles, although no universally referred to set of principles was apparent. Study descriptions, participant information, key findings and implications of these 123 studies are presented in a Additional File 3. Of the 123 studies, 60 primarily focused on message content, 6 primarily focused on delivery, and 57 focused on both.

### Narrative summary of findings

The main findings around concepts relating to message content (type of information, use of framing and use of tailoring or targeting), and message delivery (media or mode, provider/source and frequency & dose) (see Table 2 for definitions) are summarised below. This scoping review identified studies reporting on the following 11 outcomes: message recall, messages awareness, message appeal, message preference, affect, beliefs about PA, attitudes towards PA, PA intentions, self-efficacy, motivation, and PA behaviour. These outcomes can be broadly classified as proximal, intermediate or distal (see Table 4).

**Table 4:** Working definitions of relevant outcomes

Outcomes	The effects/impacts of the message
*Proximal* [32]	Immediate impacts e.g. awareness and recall of the message.
*Intermediate* [32]	Short term impacts e.g. intent, motivation, self-efficacy and knowledge.
*Distal* [32]	Medium- and long-term impacts/outcomes e.g. physical activity behaviour(s).

### Children and young people

#### Message content

Evidence from qualitative research [33, 34], existing reviews [17, 35] and experimental research [36] supports the use of messages targeting affective outcomes and highlighting the social and mental health benefits of being physically active (e.g. PA is fun and cool) in this population. Although one experimental study found no advantage for gain-framed messages over loss-framed in encouraging parent’s support for child PA [37], evidence from existing reviews and qualitative research generally supports the use of gain-framed messages when focusing on children or their parents [17, 33, 38]. Messages targeting cognitive antecedents of PA specified by Theory of Planned Behaviour (e.g. attitude) also had positive effects on intentions to exercise [39].

Mass media campaigns targeting children and young people (namely the VERB [40] and WIXX [41] campaigns) have had promising effects on PA [42] and on campaign recall and awareness [41, 43, 44]. Following the success of the social marketing-based VERB campaign, a 10-year review was published on how the campaign had informed other campaigns [45]. They found evidence of numerous program planners having aspired to follow the VERB approach, but few had taken advantage of the full capabilities of social marketing principles to bring about changes in PA behaviour.

#### Message delivery

Qualitative research suggests young people would prefer to hear messages from adults other than general practitioners (GPs) or teachers whom they felt “lectured” by [33]. Rather, formative evidence supports delivering PA messages to young people through mass media, websites and smartphone apps [17]. Experimental and survey evidence also exists to support the use of video messages over static images when targeting motivation and message recall [46, 47].

### Adults

#### Message content

Experimental evidence focusing on “how much” information (e.g. 150 min each week, 30 min × 5 days a week, or 10,000 steps each day) does not clearly support one ‘amount’ or dose as the most effective message [48, 49]. However, evidence does exist to suggest presenting 150 min each week as a minimum threshold may be damaging to perceived benefits of shorter bouts of PA [50], and that adult populations (in a national survey) show high willingness to increase PA by short bouts (i.e. 10 min per day) [51].

Overall, existing evidence from formative research and existing reviews supports the use of gain-framed messages when targeting various outcomes for adults [10, 17, 52–54]. Some experimental evidence from studies comparing gain- and loss- framed message content in trial designs have found no significant difference in effectiveness on proximal or distal outcomes in young adults [55–58], adults over 55 years [56], inactive colorectal cancer survivors [59], or in community dwelling individuals with multiple sclerosis [60]. However, an advantage for gain-framed messages over loss-framed messages has been found in general adult populations [10, 61, 62], university students [63], overweight females [64], sedentary adults [65], and cardiac rehabilitation patients [66] on outcomes including attitude, exercise intentions and PA behaviour. It also appears the effect of framing may depend on the type of outcome emphasised and on the individual’s need for cognition (i.e. an individual’s tendency to enjoy activities that require thinking) [67], or on an individual’s emotional risk perception [68]. Messages involving threat-based information or forceful language appear to be ineffective or may even have detrimental effects on PA and PA-related outcomes such as intentions, motivation and affect [69–72].

Evidence from an existing scoping review suggests mental health benefits are less frequently focused on than physical health benefits in PA messages [18]. Despite a lack of focus, evidence from existing reviews, qualitative research and and experimental research supports the use of messages highlighting short-term social (e.g. PA an opportunity to connect with others) and mental (e.g. improved mood and energy levels) health benefits in university students [73], adults [74–77] and specifically in active adults [78] when targeting various outcomes including motivation and self-efficacy. Experimental evidence identified does not support the use of appearance-based messages to improve PA intentions or attitudes in young adults [79, 80].

This scoping review identified more evidence from experimental studies demonstrating a benefit of messaging tailoring (see Table 2) in improving PA behaviour, self-efficacy and feelings towards PA [10, 81–83] than no benefit [26]. Experimental evidence also supports the use of tailoring over simple personalisation of a generic message when targeting PA behaviour [81], and no evidence was found to support the use of non-tailored messages over tailored messages. Qualitative research and existing reviews support the use of psychological theory to help identify behavioural determinants that messages can be tailored to [10, 15]. This scoping review identified a number of determinants by which messages could be tailored to, namely: Stage of Change (as described in Transtheoretical Model) [10, 84], social support needs [84–86] and self-efficacy [10, 28]. The increasing potential for intervention designers to create individually tailored messages due to advancements in technology is also apparent in the evidence base [30, 87].

The evidence supports the use of messages targeted to specific demographics, such as women [88, 89] or young healthy adults [56] to improve outcomes such as attitudes and intentions. Further, the importance of identifying and targeting to more specific population subgroups (such as education level, physical activity level, intention to be active, attitudes towards PA and perceived benefits of PA) beyond traditional demographics (such as age and gender) was evident across multiple studies and study types identified in this scoping review [25, 29, 53, 80, 82, 90–92].

Previous reviews support the use of practical advice and “how to” information in PA messages [10, 16, 74]. Existing evidence also highlights the importance of including information that is relevant to the target audience and using formative research to highlight what the specific focus of messages should be. For example, qualitative research suggests messages for women may address identified barriers to women such as poor body image [73, 93]. Mixed findings were found on the use of descriptive norm information (e.g. information about prevalence of PA amongst peers) on intentions and PA behaviour in adults [94–97].

One study found the use of spouse’s health risk information to be promising in promoting PA in middle-aged adults [98], and another found active women had higher confidence in response to reading information that PA was preventive of heart disease compared to breast cancer [99]. Potentially adverse effects of PA messages were identified in only one study, where recipients who viewed exercise-related messages consumed more calories post-message than those in control group [100].

Numerous existing reviews focusing on mass media campaigns targeting mixed adult populations were found in this scoping review [9, 12, 13, 19, 32, 101–103] as well as numerous evaluations of single mass media campaigns [47, 104–107]. Mixed findings were found for effects of campaigns on proximal outcomes (e.g. awareness and campaign recall) and intermediate outcomes (e.g. intention to be active), but generally campaigns had less of an effect on intermediate outcomes than on proximal. Campaign effects on distal outcomes such as PA behaviour itself were modest and inconsistent, with few campaigns reporting increases. However, mass media campaigns specifically targeting walking have had positive effects on awareness [108–112], attitudes [111, 113, 114] and levels of walking [108–111].

The evidence supports the use of social marketing principles (e.g. branding and promotional strategies) in the development of mass media campaigns [40, 45, 115], and suggests that interventions which use campaign building principles or social marketing benchmarks (e.g. formative research, audience segmentation and channel placement) are more successful in bringing about behaviour change than those which do not use these principles [116, 117].

#### Message delivery

The internet was found to be a common source of PA information in a general adult population [118], and interventions using the internet as a method of message delivery (e.g. email) have had promising results [119]. In terms of provider or messenger, the evidence from formative research supports the delivery of PA messages through peers [28, 53, 74] in a general adult population. In terms of media or mode of message (see Table 2), the general public find guideline documents unappealing [120], and the evidence from existing reviews and qualitative research supports the use of commercial style messages [77, 120]. Mobile phone text messages have also been successfully used in PA messaging interventions identified in this review [85, 121].

In terms of frequency and dose, when staff and students in UK universities received text messages on top of regular PA promotion emails, PA levels decreased significantly more than in the group that received emails only [122]. In young adults, evidence supports sending short messages [73] at times where there is opportunity to act on them (e.g. near morning or afternoon work break) [123], and a maximum of 2 messages per day [84]. Lastly, relating to media or mode of delivery, some experimental evidence exists to support the use of images in social media posts promoting PA [124].

In terms of mass media campaigns, qualitative research, experimental research and existing reviews identified in this scoping review support the use of multiple modalities (e.g. TV and billboard) of message delivery [47], the use of messages focusing on mental and social health benefits [125], and working with local partnerships to provide opportunities for the behaviours promoted in the campaign [126]. Longitudinal evidence following campaign effects has shown disparaities between high and low socio-economic status and between majority and minority ethnic groups, highlighting the importance of considering social inequalities when designing, implementing and evaluating mass media campaigns [127].

### Older adults

#### Message content

Experimental research in older adults supports the use of gain-framed messages over loss-framed messages in bringing about improvements in motivation and PA levels [55, 128, 129]. Qualitative evidence exists to suggest messages to older adults should highlight the short term social and mental health benefits of PA (e.g. feeling relaxed and connecting with others) [74, 130]. Messages promoting mental imagery (e.g. encouraging older adults to imagine themselves walking) may also be a promising approach to improve PA behaviour [131].

#### Message delivery

Qualitative research in older adults has found that this population have difficulty digesting technical language (e.g. ‘cardiovascular’ rather than ‘heart’) [74], and dislike the format of existing PA guideline documents [130]. Qualitative evidence also suggests older adults value messages from health care professionals (HCPs) and peers [74, 132].

### Pregnant women

#### Message content

Qualitative research found that pregnant women with Gestational Diabetes want to feel confident about being physically active during pregnancy, and that they would like practical information on safe physical activities they can take part in [133]. Empirical research suggests that appearance and health based messages were equally ineffective at improving intentions to exercise post-partum [134], but that persuasive messages grounded in Theory of Planned Behaviour resulted in significantly greater improvements in PA related outcomes than control [135].

#### Message delivery

Qualitative research supports message delivery through credible sources such as HCPs in pregnant women [133]. A randomised controlled trial found that pregnant women who received 6 PA messages/week had greater decreases in PA and increases in sedentary time than those who received fewer messages [136].

### Disabled people

#### Message content

A commentary on research conducted in disabled people supports messages promoting short term affective outcomes of PA (e.g. PA makes you feel good, do what you enjoy) [137]. Qualitative research with disabled people and their carers has highlighted the importance of acknowledging the heterogeneity of disabilities and conducting formative research to determine appropriate message content and delivery methods [138]. Qualitative research with parents of disabled children supports the use of messages including targeted information, inclusive images that promote belongingness, and messages providing self-regulatory tools [139]. One study conducted with community dwelling men and women with spinal cord injury found greater effects on proximal and intermediate outcomes following loss-framed messages targeting psychological health than gain-framed messages [140].

#### Message delivery

In parents of disabled children, qualitative research has revealed that preferred PA message providers are reliable and credible organisations, other parents [37], and the school [138]. Role models (e.g. coaches or mentors), doctors, psychologists, physiotherapists, social workers and peers have also been highlighted as important messengers for disabled people [137, 138]. Community dwelling people with spinal cord injury stated preference messages delivered via the internet and via HCPs [132].

### Gaps in the literature

**Fig. 2:**
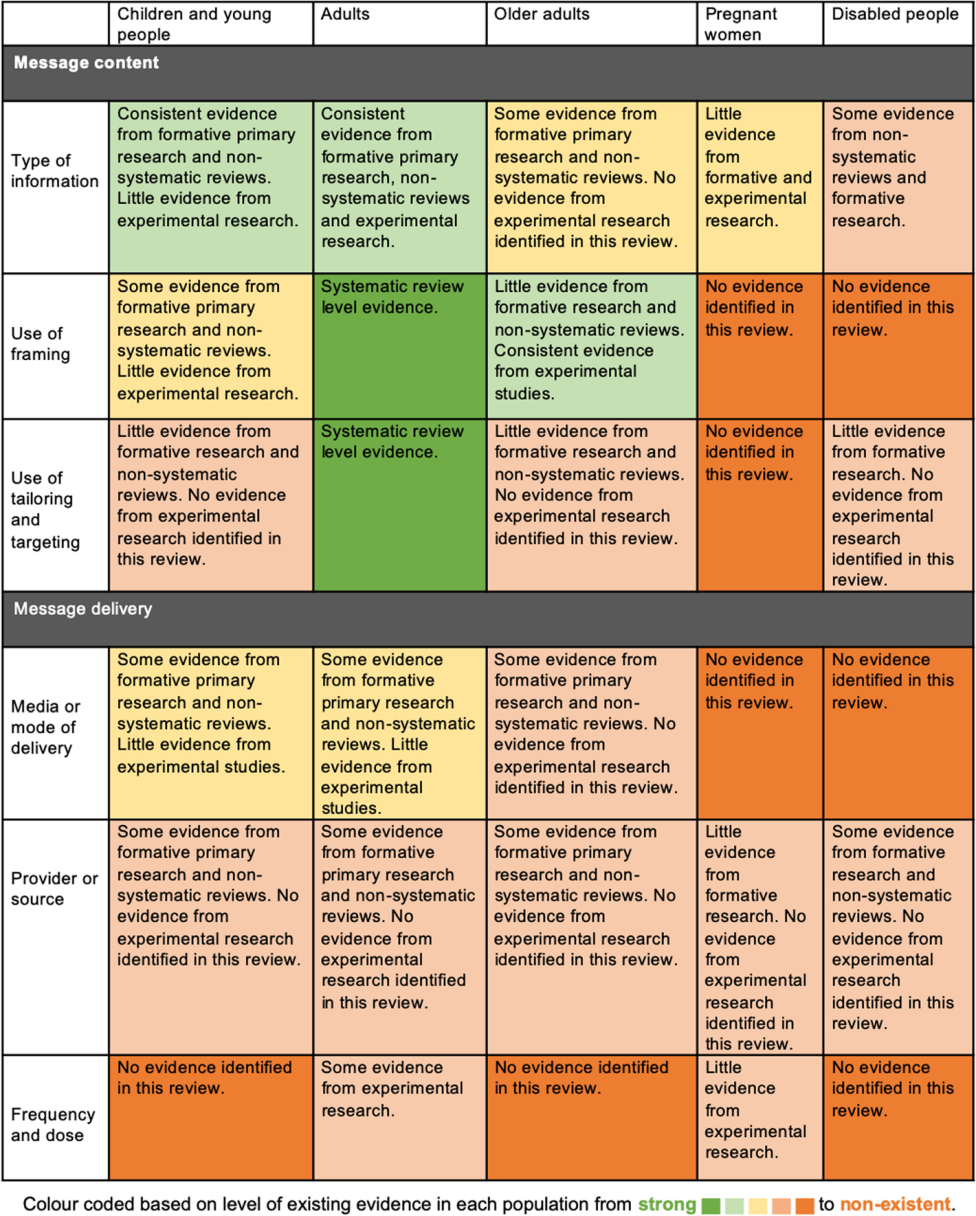
Matrix displaying where research evidence relating to physical activity messaging has accumulated and where gaps lie

With the findings from studies summarised above, it is also helpful to consider an overview of where evidence has and has not accumulated on the topic of PA messaging. Overall, studies more frequently focused on aspects of message content than on aspects of message delivery, and on adults more than other populations. A matrix displaying where evidence has accumulated and where evidence is lacking (based on the studies reviewed) is displayed in Fig. 2.

## Discussion

### Summary of principal findings

This scoping review aimed to map the literature on PA messaging and identify key research gaps. We found that PA messaging is complex and multidimensional in nature, with numerous concepts to consider when creating or evaluating messages. The extent to which each individual concept has been researched across different populations is variable and for many concepts there is no clear consensus on how to optimally design or deliver PA messages. However, the review has successfully mapped where evidence has accumulated and where clear gaps exist. Where consistent evidence does exist, it suggests that PA messages should be gain-framed, should highlight short-term outcomes (specifically relating to social and mental health), message content should be tailored or targeted, and formative research, psychological theory and/or social marketing principles should be used in message development.

### Comparison with literature and plausible explanations for findings

Our principal findings agree with those from another recent scoping review [18] that reviewed PA communication efforts solely in the USA from 1995 to 2015. The authors of that review found that 68% of messages were grounded in theory with the majority of communication research being conducted in adult populations [18]. They also recommended that PA campaigns should use visual content such as videos, content targeted to specific populations, and multiple modalities to deliver messages [18]. Our scoping review builds on these findings by providing an up to date overview of PA messaging research evidence from multiple countries across the globe and making recommendations for population groups aligning with PA for health guidelines (see Table 5 below). Our review also supports findings from a systematic review of different approaches to PA message construction [10]. In line with our findings, Latimer et al., found a benefit for gain-framed messages over loss-framed messages, found that message tailoring is important for success, and found messages targeting the psychological determinant of self-efficacy to be beneficial but concluded that overall understanding of PA messaging was lacking [10].

**Table 5:** Physical activity message recommendations based on summary of findings

Guideline group	Physical activity message recommendations
Children and young people	• Messages to this population should be framed positively, highlighting the benefits of physical activity. Specifically, messages should highlight the social and acute affective benefits of physical activity, for example, “physical activity is fun”. • Messages to this population should be delivered via engaging modes such as videos and should be delivered through informal sources such as smartphone apps or the media.
Adults	• Messages to this population should be framed positively, with specific focus on social and mental health benefits of physical activity, for example, “physical activity makes you feel good”. • Messages should be brief and should avoid threat-based language. To the general adult population, informal modes of delivery are encouraged such as through the media. • In clinical populations, messages should be delivered through health care professionals.
Older adults	• Messages to this population should be framed positively, with specific focus on social and mental health benefits of physical activity, for example, “physical activity is an opportunity to connect with others”. • Messages delivered through health care professionals are likely to be well-received
Pregnant women	• Messages to this population should include clear and practical information on physical activity during pregnancy, for example, messages could include examples of safe exercises. • Messages should be delivered through credible sources such as health care professionals.
Disabled people	• Messages to this population should highlight short-term affective benefits of physical activity and should use inclusive images. • Messages should be delivered through credible organisations, health care professionals and social workers.

Evidence from this scoping review supports the use of gain-framed messages. Message framing originates from Prospect Theory, which suggests individuals will respond differently to factually equivalent messages depending on whether they are worded to highlight benefits or consequences [141]. The benefit of gain-framing over loss-framing in promoting PA may be partially due to the greater ability or likelihood of a gain-framed messages to include information targeting psychological determinants of PA. For example, it would be difficult to construct a loss-framed message that aimed to improve an individual’s self-efficacy. This links with the finding that messages are more promising when grounded in psychological theory. This is not surprising, as the importance of using theory in the development of public health interventions is widely recognised [142] although we acknowledge this is a currently debated topic in the literature [143]. The evidence supporting the use of tailoring or targeting in PA messaging is also intuitive. It is believed that customising a message increases message salience [144], which leads to greater information processing and behaviour change [145, 146].

In a field where the physical long-term health benefits of taking part in PA are often at the forefront of epidemiology and subsequent communications, an important finding of this scoping review is that evidence supports the use of messages highlighting short-term outcomes, particularly those relating to mental and social health. This finding may relate to social marketing, which involves applying marketing techniques to influence human behaviour for social good and to improve health outcomes [147]. Social marketing involves presenting a product (in this case PA) in exchange for a cost (in this case somebody’s time, energy, or other potential resources). To market such a product effectively to an individual, it is important we make it as appealing as possible. It seems logical then that an individual may respond better to what they can get out of ‘buying into’ this product immediately (e.g. feel good, have more energy, spend time with others) than what they may get out of it later (e.g. reduced risk of cardiovascular disease later in life). An individual may know that eating cake regularly could increase their risk of weight gain and negatively affect their health, but they may continue to do so for the immediate enjoyment of eating cake. Social marketing therefore highlights the importance of utilising affective and emotional responses to make a ‘product’ more appealing [147], and this aligns with creating messages that depict PA as, for example, fun, enjoyable, or an opportunity to spend time with loved ones.

### Future research and implications

Our findings show an increasing interest in the area of PA messaging, with almost half of the studies identified published in the past 5 years. In terms of future research, there is a need for instructive studies with appropriate evaluative designs to systematically isolate and test individual components of message content and delivery for effectiveness, and thus develop our understanding of optimal PA content and delivery in different population subgroups. Such research efforts should prioritise PA the messaging concepts identified in this review as having little or no evidence focusing on them (e.g. message dose and frequency in all populations, see Table 5). In areas where evidence has accumulated but has not yet been synthesised (e.g. type of information in adults, see Table 5), systematic reviews with meta-analyses are also warranted. Qualitative research and mediator analysis to help gain a greater understanding of the specific mechanisms by which existing PA messages work (i.e. which psychological determinants are affected by PA messages), or which outcomes future PA messages should target is also required to enhance our understanding of PA messaging. Also, although we attempted to identify commonly used theories from included studies, numerous potentially important theories were not discussed in this review (e.g. Knowledge Gap Hypothesis and Elaboration Likelihood Model). Therefore, to further enhance our understanding of PA messaging, a specific review of the role of theories and the extent to which they have been used in PA messaging using existing guidance [148] may also be warranted. Lastly, due to the complex and multidimensional nature of PA messaging highlighted in this review, there is a need to organise and conceptualise the area of PA messaging to encourage further understanding of, and application in, this area.

The findings of this research are timely as they could inform the dissemination of newly developed, or updated guidelines to various populations [31, 149–151]. At current, the World Health Organistion (WHO) are updating global PA guidelines, and these findings may aid their communication and dissemination plans. In the UK specifically, the Updated Chief Medical Officers’ (CMO) guidelines (released on September 9th 2019) state that a Communications Working Group is being established to advise approaches to communicating PA recommendations and related messages to the wider public [31]. Based on key findings, recommendations which could be used by such working groups to aid development of PA messages to each guideline group are presented below (Table 5).

### Strengths and limitations

This scoping review is the first to attempt to provide an overview of available evidence on PA messaging from across multiple countries. A key strength of this scoping review is its inclusivity of a range of study designs, allowing us to provide a more comprehensive overview of the evidence base. Further strengths of this scoping review include the use of established protocol [20–22], and presentation of findings by key concepts and population groups. This review has produced meaningful findings which may aid dissemination of PA guidelines (such as the under-development new WHO Global PA guidelines or the newly released UK PA guidelines for health [31]) and related information to various population subgroups.

Due to the nature of scoping reviews, we did not appraise the quality of evidence included. We are also unable to comment on the effectiveness of different messaging techniques as the heterogeneous nature of included studies do not allow for meta-analysis, and indeed that is not the aim of a scoping review [20]. Rather, we have presented a descriptive account of available research. Another limitation of this scoping review is that only titles were searched due to the time and resource constraints. This limitation means that some relevant studies were missed. For example, although our review identified the Canadian campaign ParticipACTION [46], not all publications on this campaign were identified due to the limitations of our search terms [152]. Indeed, given the nature of PA messaging, it could be argued that it is impossible for one review to completely capture all of the available evidence in this area. However, a substantial body of literature was generated (123 studies); almost double the number identified in a previous scoping review of PA communication in the USA alone [18]. We are confident we have included a range of studies that adequately provide an overview of the PA messaging evidence base, and which is sufficient in addressing our aims. Finally, there are likely lessons to be learned from other forms of PA communication (e.g. one-to-one counselling) however, these were deemed to be outside the scope of this study and therefore not included. Isolating PA messaging to the public from other forms of communication was necessary to focus our study and develop our understanding of PA messaging and its application in this area.

## Conclusion

While it is unlikely to address global inactivity on its own, PA messaging may play a significant role in targeting individual factors in a systems approach to improve PA population levels, but is a complex and multidimensional concept. We present a synthesis of the PA messaging evidence from across the globe, highlighting key areas where evidence has accumulated and where gaps exist. We provide recommendations for PA messaging to different population groups. Headline findings include support for the use of gain-framed messages highlight short-term mental and social health outcomes, tailored or targeted messages, and messages grounded in psychological theory or social marketing principles. Further instructive research is required to understand how to optimally message PA information to different populations.

## Supplementary information

**Additional file 1.** PRISMA Scoping Review Checklist.

**Additional file 2.** Search terms and strategy.

**Additional file 3.** Supplementary datasheet of included studies.

## Data Availability

Details of reviewed articles are available in Additional File 3.

## Abbreviations

PA: physical activity
HCP: health care professional
GP: general practitioner
CMO: chief medical officers
UK: United Kingdom
USA: United States of America

## References

[1] Lee IM, Shiroma EJ, Lobelo F, Puska P, Blair SN, Katzmarzyk PT (2012). Effect of physical inactivity on major non- communicable diseases worldwide: an analysis of burden of disease and life expectancy. Lancet..

[2] Guthold R, Stevens GA, Riley LM, Bull FC (2018). Worldwide trends in insufficient physical activity from 2001 to 2016: a pooled analysis of 358 population-based surveys with 1·9 million participants. Lancet Glob Health.

[3] Rutter H, Savona N, Glonti K, Bibby J, Cummins S, Finegood DT (2017). The need for a complex systems model of evidence for public health. Lancet.

[4] Peters D (2014). The application of systems thinking in health: why use systems thinking?. Health Res Policy Syst.

[5] Hawe P, Shiell A, Riley T (2009). Theorising interventions as events in systems. Am J Community Psychol.

[6] Friel S, Pescud M, Malbon E, Lee A, Carter R, Greenfield J (2017). Using systems science to understand the determinants of inequities in healthy eating. PLoS One.

[7] Rutter H, Cavill N, Bauman A, Bull F (2019). Systems approaches to global and national physical activity plans. Bull World Health Organ.

[8] Sallis JF, Bauman A, Pratt M (1998). Environmental and policy interventions to promote physical activity.

[9] Cavill N, Bauman A (2004). Changing the way people think about health-enhancing physical activity: do mass media campaigns have a role?. J Sports Sci.

[10] Latimer AE, Brawley L, Bassett R. A systematic review of three approaches for constructing physical activity messages: What messages work and what improvements are needed? Int J Behav Nutr Phys Act. 2010. 10.1186/1479-5868-7-36 PMC2885311 20459779

[11] Scottish Government (2012). Scottish health survey: Main report.

[12] Brown DR, Soares J, Epping JM, Lankford TJ, Wallace JS, Hopkins D (2012). Stand-alone mass media campaigns to increase physical activity:a community guide updated review: a community guide updated review. Am J Prev Med.

[13] Finlay S-J, Faulkner G (2005). Physical activity promotion through the mass media: inception, production, transmission and consumption. Prev Med.

[14] Leavy J, Rosenberg M, Bull F, Corti BG, Shilton T, Maitland C (2011). Effects of Find Thirty every day: Cross sectional findings from a Western Australian population wide mass media campaign 2008–2010. J Sci Med Sport.

[15] Berry TR, Latimer-Cheung AE (2013). Overcoming challenges to build strong physical activity promotion messages. Am J Lifestyle Med.

[16] Brawley LR, Latimer AE (2007). Physical activity guides for Canadians: messaging strategies, realistic expectations for change, and evaluation. Can J Public Health.

[17] Latimer-Cheung AE, Rhodes R, Kho ME, Tomasone JR, Gainforth HL, Kowalski K (2013). Evidence-informed recommendations for constructing and disseminating messages supplementing the new Canadian Physical Activity Guidelines. BMC Public Health.

[18] Bergeron CD, Tanner AH, Friedman DB, Zheng Y, Schrock CS, Bornstein DB (2019). Physical activity communication: a scoping review of the literature. Health Promot Pract.

[19] Leavy JE, Bull FC, Rosenberg M, Bauman A (2011). Physical activity mass media campaigns and their evaluation: a systematic review of the literature 2003-2010. Health Educ Res.

[20] Arksey H, Malley L (2005). Scoping studies: towards a methodological framework. Int J Soc Res Methodol.

[21] Levac D, Colquhoun H, Brien KK. Scoping studies: advancing the methodology. Implement Sci. 2010;5(1) <xocs:firstpage xmlns:xocs=""/>. 10.1186/1748-5908-5-69 PMC2954944 20854677

[22] Tricco AC, Lillie E, Zarin W, O’Brien KK, Colquhoun H, Levac D (2018). PRISMA extension for scoping reviews (PRISMA-ScR): checklist and explanation. Ann Intern Med.

[23] US Department of Health and Human Services. Healthy People 2010 Final Review. p. 2010.

[24] Michie S, VMM S, West R. The behaviour change wheel: a new method for characterising and designing behaviour change interventions. Implement Sci. 2011;6. 10.1186/1748-5908-6-42 PMC3096582 21513547

[25] Yap T, Davis S (2008). Physical activity: the science of health promotion through tailored messages. Rehabil Nurs.

[26] Martinez JL, Duncan LR, Rivers SE, Latimer AE, Salovey P (2013). Examining the use of message tailoring to promote physical activity among medically underserved adults. J Health Psychol.

[27] Yan A (2015). MHealth text messaging for physical activity promotion in college youth: a participatory approach. Ann Behav Med.

[28] Marmo J (2013). Applying social cognitive theory to develop targeted messages: college students and physical activity. West J Commun.

[29] Berry TR (2016). Changes in implicit and explicit exercise-related attitudes after reading targeted exercise-related information. Psychol Sport Exerc.

[30] Conroy DE, Hojjatinia S, Lagoa CM, Yang C-H, Lanza ST, Smyth JM (2019). Personalized models of physical activity responses to text message micro-interventions: a proof-of-concept application of control systems engineering methods. Psychol Sport Exerc.

[31] Scottish Government (2019). UK chief medical Officers’ physical activity guidelines.

[32] Bauman A, Chau J (2009). The role of media in promoting physical activity. J Phys Act Health.

[33] Nicholson L (2012). Development of key themes for physical activity promotion for the early years, children and young people.

[34] Bellows L, Spaeth A, Lee V, Anderson J (2013). Exploring the use of storybooks to reach mothers of preschoolers with nutrition and physical activity messages. J Nutr Educ Behav.

[35] Alberga A, Fortier M, Bean C, Freedhoff Y (2019). Youth get a D+ grade in physical activity: how can we change public health messages to help reverse this trend?. Appl Physiol Nutr Metab.

[36] Sirriyeh R, Lawton R, Ward J (2010). Physical activity and adolescents: an exploratory randomized controlled trial investigating the influence of affective and instrumental text messages. Br J Health Psychol.

[37] Bassett-Gunter R, Stone R, Jarvis J, Latimer-Cheung A (2017). Motivating parent support for physical activity: the role of framed persuasive messages. Health Educ Res.

[38] Jarvis J, Gainforth H, Latimer-Cheung A (2014). Investigating the effect of message framing on parents’ engagement with advertisements promoting child physical activity. Int Rev Public Nonprofit Market.

[39] Hill C, Abraham C, Wright DB (2007). Can theory-based messages in combination with cognitive prompts promote exercise in classroom settings?. Soc Sci Med.

[40] Wong F, Huhman M, Heitzler C, Asbury L, Bretthauer-Mueller R, McCarthy S (2004). VERB - a social marketing campaign to increase physical activity among youth. Prev Chronic Dis.

[41] Bélanger-Gravel A, Gauvin L, Lagarde F, Laferté M (2014). Initial recall and understanding of a multimedia communication campaign to promote physical activity among tweens: a process evaluation study. Prev Med.

[42] Huhman M, Potter LD, Wong FL, Banspach SW, Duke JC, Heitzler CD (2005). Effects of a mass media campaign to increase physical activity mmong children: year-1 results of the VERB campaign.(Author Abstract). Pediatrics.

[43] Bélanger-Gravel A, Cutumisu N, Gauvin L, Lagarde F, Laferté M (2017). Correlates of initial recall of a multimedia communication campaign to promote physical activity among tweens: the WIXX campaign. Health Commun.

[44] Bélanger-Gravel A, Cutumisu N, Lagarde F, Laferté M, Gauvin L (2017). Short-term impact of a multimedia communication campaign on Children’s physical activity beliefs and behavior. J Health Commun.

[45] Huhman M, Kelly R, Edgar T (2017). Social marketing as a framework for youth physical activity initiatives: a 10-year retrospective on the legacy of CDC’s VERB campaign. Curr Obes Rep.

[46] Deshpande S, Berry TR, Faulkner GEJ, Latimer-Cheung AE, Rhodes RE, Tremblay MS (2015). Comparing the influence of dynamic and static versions of Media in Evaluating Physical-Activity-Promotion ads. Soc Marketin Quart.

[47] Peterson M, Chandlee M, Abraham A (2008). Cost-effectiveness analysis of a statewide media campaign to promote adolescent physical activity. Health Promot Pract.

[48] Murtagh EM, Archibald K, Doherty A, Mutrie N, Breslin G, Lambe B, et al. 150 minutes per week or 30 minutes on 5 days? The effect of brief advice about physical activity recommendations on moderate-to-vigorous activity of inactive adults. In: Limerick Uo, editor. 2014.

[49] Pal S, Cheng C, Ho S (2011). The effect of two different health messages on physical activity levels and health in sedentary overweight, middle-aged women. BMC Public Health.

[50] Knox ECL, Webb OJ, Esliger DW, Biddle SJH, Sherar LB (2014). Using threshold messages to promote physical activity: implications for public perceptions of health effects. Eur J Public Health.

[51] Miyachi M, Tripette J, Kawakami R, Murakami H (2015). “+10 min of physical activity per day”: Japan is looking for efficient but feasible recommendations for its population. J Nutr Sci Vitaminol.

[52] Adams R (2000). Shown to be useful. Survey shows positive results for first-ever set of national physical activity guidelines. Can Fam Physician.

[53] Black DR, Blue CL, Kosmoski K, Coster DC (2000). Social marketing: developing a tailored message for a physical activity program. Am J Health Behav.

[54] Mendez RDR, Rodrigues RCM, Spana TM, Cornélio ME, Gallani MCBJ, Pérez-Nebra AR (2012). Validation of persuasive messages for the promotion of physical activity among people with coronary heart disease. Rev Lat Am Enfermagem.

[55] Notthoff N, Carstensen LL (2014). Positive messaging promotes walking in older adults. Psychol Aging.

[56] Berry TR, Carson V (2010). Ease of imagination, message framing, and physical activity messages. Br J Health Psychol.

[57] Kin-Kit L, Sheung-Tak C, Fung HH (2014). Effects of message framing on self-report and accelerometer-assessed physical activity across age and gender groups. J Sport Exerc Psychol.

[58] Bassett-Gunter RL, Latimer-Cheung AE, Martin Ginis KA, Castelhano M (2014). I spy with my little eye: cognitive processing of framed physical activity messages. J Health Commun.

[59] Hirschey R, Lipkus I, Jones L, Mantyh C, Sloane R, Demark-Wahnefried W (2016). Message framing and physical activity promotion in colorectal cancer survivors.(Report). Oncol Nursing Forum.

[60] Lithopoulos A, Bassett-Gunter RL, Martin Ginis KA, Latimer-Cheung AE (2017). The effects of gain- versus loss-framed messages following health risk information on physical activity in individuals with multiple sclerosis. J Health Commun.

[61] Sweet SN, Brawley LR, Hatchell A, Gainforth HL, Latimer-Cheung AE (2014). Can persuasive messages encourage individuals to create action plans for physical activity?. J Sport Exerc Psychol.

[62] van’t Riet J, RAC R, Werrij MQ, de Vries H (2010). Investigating message-framing effects in the context of a tailored intervention promoting physical activity. Health Educ Res.

[63] Jones LW, Sinclair RC, Courneya KS (2003). The effects of source credibility and message framing on exercise intentions, behaviors, and attitudes: an integration of the elaboration likelihood model and Prospect Theory1. J Appl Soc Psychol.

[64] Kozak AT, Nguyen C, Yanos BR, Fought A (2013). Persuading students to exercise: what is the best way to frame messages for Normal-weight versus overweight/Obese University students?. J Am Coll Heal.

[65] Latimer AE, Rench TA, Rivers SE, Katulak NA, Materese SA, Cadmus L (2008). Promoting participation in physical activity using framed messages: an application of prospect theory. Br J Health Psychol.

[66] McCall LA, Ginis KAM (2004). The effects of message framing on exercise adherence and health beliefs among patients in a cardiac rehabilitation program. J Appl Biobehav Res.

[67] Gallagher KM, Updegraff JA (2011). When 'fit' leads to fit, and when 'fit' leads to fat: how message framing and intrinsic vs. extrinsic exercise outcomes interact in promoting physical activity. Psychol Health.

[68] Michalovic E, Hall S, Duncan LR, Bassett-Gunter R, Sweet SN (2018). Understanding the effects of message framing on physical activity action planning: the role of risk perception and elaboration. Int J Behav Med.

[69] Kang Y, O’Donnell MB, Strecher VJ, Falk EB (2017). Dispositional mindfulness predicts adaptive affective responses to health messages and increased exercise motivation.(Report)(Author abstract). Mindfulness.

[70] Quick BL, Considine JR (2008). Examining the use of forceful language when designing exercise persuasive messages for adults: a test of conceptualizing reactance arousal as a two-step process. Health Commun.

[71] Hatchell AC, Bassett-Gunter RL, Clarke M, Kimura S, Latimer-Cheung AE (2013). Messages for men: the efficacy of EPPM-based messages targeting Men's physical activity. Health Psychol.

[72] Brengman M, Wauters B, Macharis C, Mairesse O (2010). Functional effectiveness of threat appeals in exercise promotion messages. Psicologica.

[73] Reese JM, Joseph RP, Cherrington A, Allison J, Kim Y-I, Spear B (2017). Development of participant-informed text messages to promote physical activity among African American women attending college: a qualitative mixed-methods inquiry. J Transcult Nurs.

[74] Nicholson L (2012). Development of key themes for physical activity promotion for adults - summary report.

[75] Howle TC, Dimmock JA, Ntoumanis N, Chatzisarantis NLD, Sparks C, Jackson B (2017). The impact of Agentic and communal exercise messages on Individuals' exercise class attitudes, self-efficacy beliefs, and intention to attend. J Sport Exerc Psychol.

[76] Groshong L, Stanis SAW, Kaczynski AT, Hipp JA, Besenyi GM (2017). Exploring attitudes, perceived norms, and personal agency: insights into theory-based messages to encourage park-based physical activity in low-income urban neighborhoods. J Phys Act Health.

[77] Berry T (2017). Rethinking how physical activity messages are thought about: implications for successful promotion. WellSpring..

[78] Hevel DJ, Amorose AJ, Lagally KM, Rinaldi-Miles A, Pierce S (2019). Testing the effects of messaging on physical activity motivation in active and non-active adults. Psychol Sport Exerc.

[79] Rhodes R, Courneya K (2000). Effects of a health-based versus appearance-based persuasive message on attitudes towards exercise: testing the moderating role of self-monitoring. J Soc Behav Pers.

[80] Berry TR, Jones KE, McLeod NC, Spence JC (2011). The relationship between implicit and explicit believability of exercise-related messages and intentions. Health Psychol.

[81] Bull FC, Kreuter MW, Scharff DP (1999). Effects of tailored, personalized and general health messages on physical activity. Patient Educ Couns.

[82] Latimer AE, Rivers SE, Rench TA, Katulak NA, Hicks A, Hodorowski JK (2008). A field experiment testing the utility of regulatory fit messages for promoting physical activity. J Exp Soc Psychol.

[83] Quintiliani LM, Campbell MK, Bowling JM, Steck S, Haines PS, DeVellis BM (2010). Results of a randomized trial testing messages tailored to participant-selected topics among female college students: physical activity outcomes. J Phys Act Health.

[84] Yan A, Stevens P, Wang Y, Weinhardt L, Holt C, O'Connor C (2015). MHealth text messaging for physical activity promotion in college youth: a participatory approach. Ann Behav Med.

[85] Kinnafick FE, Thogersen-Ntoumani C, Duda JL. The effect of need supportive text messages on motivation and physical activity behaviour. 2016;39(4). 10.1007/s10865-016-9722-1 PMC4942483 26915963

[86] Bailis DS, Ashley Fleming J, Segall A (2005). Self-determination and functional persuasion to encourage physical activity. Psychol Health.

[87] Op Den Akker H, Cabrita M, Op Den Akker R, Jones VM, Hermens HJ (2015). Tailored motivational message generation: A model and practical framework for real-time physical activity coaching. J Biomed Inform.

[88] Leone LA, Campbell MK, Allicock M, Pignone M (2012). Colorectal Cancer screening and physical activity promotion among obese women: an online evaluation of targeted messages. J Health Commun.

[89] Thai CL, Taber JM, Oh A, Segar M, Blake K, Patrick H (2019). "keep it realistic": reactions to and recommendations for physical activity promotion messages from focus groups of women. Am J Health Promot.

[90] Cheval B, Sarrazin P, Isoard-Gautheur S, Radel R, Friese M (2015). Reflective and impulsive processes explain (in)effectiveness of messages promoting physical activity: a randomized controlled trial. Health Psychol.

[91] Leahy J, Shugrue M, Daigle J, Daniel H (2009). Local and visitor physical activity through media messages: a specialized benefits-based Manageent application at Acadia National Park. J Park Recreat Adm.

[92] Berry T. Who’s even interested in the exercise message? Attentional bias for exercise and sedentary-lifestyle related words. J Sport Exerc Psychol. 2006;28(1).

[93] Segar M, Taber JM, Patrick H, Thai CL, Oh A. Rethinking physical activity communication: using focus groups to understand women’s goals, values, and beliefs to improve public health. BMC Public Health. 2017;17. 10.1186/s12889-017-4361-1 PMC5437577 28521756

[94] Priebe CS, Spink KS (2012). Using messages promoting descriptive norms to increase physical activity. Health Commun.

[95] Priebe CS, Spink KS (2015). Less sitting and more moving in the office: using descriptive norm messages to decrease sedentary behavior and increase light physical activity at work. Psychol Sport Exerc.

[96] van Bavel R, Esposito G, Baranowski T. Is anybody doing it? An experimental study of the effect of normative messages on intention to do physical activity.(Survey). BMC Public Health. 2014;14(1). 10.1186/1471-2458-14-778 PMC4139606 25082214

[97] Crozier AJ, Taylor KL (2019). An exploratory study examining the interactive effect of descriptive norm and image appeal messages on Adults' physical activity intentions: a test of deviation regulation theory. J Health Commun.

[98] Skapinsky KF, Persky S, Lewis M, Goergen A, Ashida S, de Heer HD (2018). Heart disease risk information, encouragement, and physical activity among Mexican-origin couples: self- or spouse-driven change?. Transl Behav Med.

[99] Berry TR, Jones KE, Courneya KS, McGannon KR, Norris CM, Rodgers WM (2018). Believability of messages about preventing breast cancer and heart disease through physical activity. BMC Psychol.

[100] Albarracin D, Wang W, Leeper J (2009). Immediate increase in food intake following exercise messages. Obesity (Silver Spring, Md).

[101] Yun L, Ori EM, Younghan L, Sivak A, Berry TR (2017). A systematic review of community-wide media physical activity campaigns: an update from 2010. J Phys Act Health.

[102] Marcus B, Owen N, Forsyth L, Cavill N, Fridinger F (1998). Physical activity interventions using mass media, print media, and information technology. Am J Prev Med.

[103] Marshall AL, Owen N, Bauman AE (2004). Mediated approaches for influencing physical activity: update of the evidence on mass media, print, telephone and website delivery of interventions. J Sci Med Sport.

[104] Bauman AE, Bellew B, Owen N, Vita P (2001). Impact of an Australian mass media campaign targeting physical activity in 1998. Am J Prev Med.

[105] Smith BJ, Bonfiglioli CMF (2015). Physical activity in the mass media: an audience perspective. Health Educ Res.

[106] Saito Y, Oguma Y, Tanaka A, Kamada M, Inoue S, Inaji J (2018). Community-wide physical activity intervention based on the Japanese physical activity guidelines for adults: a non-randomized controlled trial. Prev Med.

[107] Peterson M, Abraham A, Waterfield A (2005). Marketing physical activity: lessons learned from a statewide media campaign. Health Promot Pract.

[108] Owen N, Bauman A, Booth M, Oldenburg B, Magnus P (1995). Serial mass-media campaigns to promote physical activity: reinforcing or redundant?. Am J Public Health.

[109] Booth M, Bauman A, Oldenburg B, Owen N, Magnus P (1992). Effects of a National Mass-Media Campaign on physical activity participation. Health Promot Int.

[110] Barnes R, Giles-Corti B, Bauman A, Rosenberg M, Bull F, Leavy J (2013). Does neighbourhood walkability moderate the effects of mass media communication strategies to promote regular physical activity?. Ann Behav Med.

[111] Leavy JE, Rosenberg M, Bauman AE, Bull FC, Giles-Corti B, Shilton T (2013). Effects of find thirty every day(R): cross-sectional findings from a Western Australian population-wide mass media campaign, 2008-2010. Health Educ Behav.

[112] Leavy JE, Rosenberg M, Bull FC, Bauman AE (2014). Who do we reach? Campaign evaluation of find thirty every day(R) using awareness profiles in a Western Australian cohort. J Health Commun.

[113] Beaudoin CE, Fernandez C, Wall JL, Farley TA (2007). Promoting healthy eating and physical activity: short-term effects of a mass media campaign. Am J Prev Med.

[114] Leavy JE, Bauman AE, Rosenberg M, Bull FC (2014). Examining the communication effects of health campaigns. SAGE Open.

[115] Buller DB (2006). Diffusion and dissemination of physical activity recommendations and programs to world populations. Am J Prev Med.

[116] Xia Y, Deshpande S, Bonates T (2016). Effectiveness of social marketing interventions to promote physical activity among adults: a systematic review. J Phys Act Health.

[117] Lankford T, Wallace J, Brown D, Soares J, Epping J, Fridinger F (2014). Analysis of physical activity mass media campaign design. J Phys Act Health.

[118] Berry TR, Spence JC, Plotnikoff RC, Bauman A (2011). Physical activity information seeking and advertising recall. Health Commun.

[119] Rhee Y, Nyquist H, Garden-Robinson J, Brunt A (2009). Promoting healthy eating and exercise through online messages. J Am Diet Assoc.

[120] Berry TR, Witcher C, Holt NL, Plotnikoff RC (2010). A qualitative examination of perceptions of physical activity guidelines and preferences for format. Health Promot Pract.

[121] Filion AJ, Darlington G, Chaput JP, Ybarra M, Haines J (2015). Examining the influence of a text message-based sleep and physical activity intervention among young adult smokers in the United States. BMC Public Health.

[122] Suggs S, Blake H, Bardus M, Lloyd S (2013). Effects of text messaging in addition to emails on physical activity among university and college employees in the UK. J Health Serv Res Policy.

[123] McCoy P, Leggett S, Bhuiyan A, Brown D, Frye P, Williams B. Text messaging: an intervention to increase physical activity among African American Participants in a Faith-Based, Competitive Weight Loss Program. Int J Environ Res Public Health. 2017;14(4). 10.3390/ijerph14040326 PMC5409538 28353650

[124] Johnston C, Davis WE (2019). Motivating exercise through social media: is a picture always worth a thousand words?. Psychol Sport Exerc.

[125] Scarapicchia TMF, Sabiston CMF, Brownrigg M, Blackburn-Evans A, Cressy J, Robb J, et al. MoveU? Assessing a social marketing campaign to promote physical activity. J Am Coll Health. 2015. 10.1080/07448481.2015.1025074 25774868

[126] Graham DJ, Graham JF (2008). Improving media campaigns promoting physical activity: the underutilized role of gender. Int J Nonprofit Volunt Sect Mark.

[127] Pena-Y-Lillo M, Lee C-J (2019). A communication inequalities approach to disparities in physical activities: the case of the VERB campaign. J Health Commun.

[128] Notthoff N, Klomp P, Doerwald F, Scheibe S (2016). Positive messages enhance older adults’ motivation and recognition memory for physical activity programmes. Soc Behav Health Perspec.

[129] Li K, Lorna N, Sheung-Tak C, Helene HF, Li K-K, Ng L (2017). Reverse message-framing effects on accelerometer-assessed physical activity among older outpatients with type 2 diabetes. J Sport Exerc Psychol.

[130] Sebastiao E, Chodzko-Zajko W, Schwingel A. The need to modify physical activity messages to better speak to older African American women: a pilot study.(Report). BMC Public Health. 2015;15(1). 10.1186/s12889-015-2317-x PMC4582709 26403196

[131] Robin N, Toussaint L, Coudevylle GR, Ruart S, Hue O, Sinnapah S (2018). Text messages promoting mental imagery increase self-reported physical activity in older adults: a randomized controlled study. J Aging Phys Act.

[132] Letts L, Martin Ginis KA, Faulkner G, Colquhoun H, Levac D, Gorczynski P (2011). Preferred methods and messengers for delivering physical activity information to people with spinal cord injury: a focus group study. Rehabil Psychol.

[133] Harrison AL, Taylor NF, Frawley HC, Shields N (2019). Women with gestational diabetes mellitus want clear and practical messages from credible sources about physical activity during pregnancy: a qualitative study. J Phys.

[134] Gaston A, Gammage K (2010). Health versus appearance messages, self-monitoring and pregnant women’s intentions to exercise postpartum. J Reprod Infant Psychol.

[135] Gaston A, Gammage K (2011). The effectiveness of a health-based message on pregnant women’s intentions to exercise postpartum. J Reprod Infant Psychol.

[136] Huberty JL, Buman MP, Leiferman JA, Bushar J, Hekler EB, Adams MA (2017). Dose and timing of text messages for increasing physical activity among pregnant women: a randomized controlled trial. Transl Behav Med.

[137] Smith B, Wightman L. Promoting physical activity to disabled people: messengers, messages, guidelines and communication formats. Disabil Rehabil. 2019;1. 10.1080/09638288.2019.1679896 31638443

[138] Jaarsma EA, Haslett D, Smith B (2019). Improving communication of information about physical activity opportunities for people with disabilities. Adapt Phys Act Q.

[139] Bassett-Gunter RL, Ruscitti RJ, Latimer-Cheung AE, Fraser-Thomas JL (2017). Targeted physical activity messages for parents of children with disabilities: a qualitative investigation of parents' informational needs and preferences. Res Dev Disabil.

[140] Bassett-Gunter RL, Martin Ginis KA, Latimer-Cheung AE (2013). Do you want the good news or the bad news? Gain- versus loss-framed messages following health risk information: the effects on leisure time physical activity beliefs and cognitions. Health Psychol.

[141] Tversky A, Kahneman D (1981). The framing of decisions and the psychology of choice. Science..

[142] Green LW, Kreuter MW (1999). Health promotion planning : an educational and ecological approach.

[143] Hagger MS, Weed M. DEBATE: Do interventions based on behavioral theory work in the real world?(Report). Int J Behav Nutr Phys Activity. 2019;16(1). 10.1186/s12966-019-0795-4 PMC6482531 31023328

[144] Kreuter M, Strecher V, Glassman B (1999). One size does not fit all: the case for tailoring print materials. Ann Behav Med.

[145] Marcus (2010). Physical Activity Intervention Studies: What We Know and What We Need to Know: A Scientific Statement From the American Heart Association Council on Nutrition, Physical Activity, and Metabolism (Subcommittee on Physical Activity); Council on Cardiovascular Disease in the Young; and the Interdisciplinary Working Group on Quality of Care and Outcomes Research (vol 114, pg 2739, 2006). Circulation.

[146] Napolitano AM, Marcus HB (2002). Targeting and tailoring physical activity information using print and information technologies. Exerc Sport Sci Rev.

[147] Hastings G. Social marketing. Third edition.. ed. Domegan C, editor. Abingdon, Oxon ; New York, NY: Abingdon, Oxon ; New York, NY : Routledge; 2018.

[148] Michie S, Prestwich A (2010). Are interventions theory-based? Development of a theory coding scheme. Health Psychol.

[149] US Department of Health and Human Services. Physical Activity Guidelines for Americans, 2nd Edition. In: Services DoHaH, editor. Washington, DC. USA.2018.

[150] Brown W, Bauman A, Bull F, Burton N (2012). Development of evidence-based physical activity recommendations for adults (18–64 years).

[151] World Health Organization. Guidelines on physical activity, sedentary behaviour and sleep for children under 5 years of age. Geneva; 2019. Contract No.: 23rd March 2020. 31091057

[152] Spence JC, Brawley LR, Craig CL, Plotnikoff RC, Tremblay MS, Bauman A (2009). ParticipACTION: awareness of the participACTION campaign among Canadian adults--examining the knowledge gap hypothesis and a hierarchy-of-effects model. Int J Behav Nutr Phys Activity.

