# Supplementary material for: Get the message? A scoping review of physical activity messaging
Source: Int J Behav Nutr Phys Act. 2020 Apr 15;17:51. doi: 10.1186/s12966-020-00954-3 (PMC7160981; doi:10.1186/s12966-020-00954-3)
Supplement: Supplementary file 2 — Additional file 2. Search terms and strategy. [file 12966_2020_954_MOESM2_ESM.docx]

**Additional File 2: Search terms and strategy**

*Supplementary Table 1: Search terms*

| **Physical activity terms** | **Messaging terms** |
| --- | --- |
| Physical activity  Exercise  Fitness | Message  Messaging  Persuasion  Information  Communication  Media  Guidelines  Recommendations  Marketing |

*Supplementary Table 2: Example search strategy (MEDLINE)*

| 1. physical activit*.ti.  2. exercise*.ti.  3. fitness.ti.  4. 1 or 2 or 3  5. messag*.ti.  6. persuas*.ti.  7. information.ti.  8. communication.ti.  9. media.ti.  10. education*.ti.  11. guideline*.ti.  12. recommendation*.ti.  13. market*.ti.  14. 5 or 6 or 7 or 8 or 9 or 10 or 11 or 12 or 13  15. 4 and 14 |
| --- |
